# Comparison of unmanned aerial vehicle imaging to ground truth walkthroughs for identifying and classifying trash sites serving as potential *Aedes aegypti* breeding grounds

**DOI:** 10.1186/s13071-025-06706-1

**Published:** 2025-03-06

**Authors:** Morgan S. Tarpenning, Juliet T. Bramante, Kavita D. Coombe, Katherine E. Woo, Andrew J. Chamberlin, Paul S. Mutuku, Giulio A. De Leo, Angelle Desiree LaBeaud, Bryson A. Ndenga, Francis M. Mutuku, Joelle I. Rosser

**Affiliations:** 1 Stanford University, Stanford, CA USA; 2 University of Washington, School of Medicine, Seattle, WA USA; 3 Division of Infectious Diseases, Stanford University, School of Medicine, Stanford, CA USA; 4 Department of Earth System Sciences and Department of Oceans, Stanford University, Hopkins Marine Institute, Stanford, CA USA; 5 Technical University of Mombasa, Mombasa, Kenya; 6 Department of Pediatrics, Division of Infectious Diseases, Stanford University, School of Medicine, Stanford, CA USA; 7 Centre for Global Health Research, Kenya Medical Research Institute, Kisumu, Kenya

**Keywords:** Aedes, Remote sensing technology, Unmanned aerial devices, Waste management

## Abstract

**Background:**

Trash piles and abandoned tires that are exposed to the elements collect water and create productive breeding grounds for *Aedes aegypti* mosquitoes, the primary vector for multiple arboviruses. Unmanned aerial vehicle (UAV) imaging provides a novel approach to efficiently and accurately mapping trash, which could facilitate improved prediction of *Ae*. *aegypti* habitat and consequent arbovirus transmission. This study evaluates the efficacy of trash identification by UAV imaging analysis compared with the standard practice of walking through a community to count and classify trash piles.

**Methods:**

We conducted UAV flights and four types of walkthrough trash surveys in the city of Kisumu and town of Ukunda in western and coastal Kenya, respectively. Trash was classified on the basis of a scheme previously developed to identify high and low risk *Aedes aegypti* breeding sites. We then compared trash detection between the UAV images and walkthrough surveys.

**Results:**

Across all walkthrough methods, UAV image analysis captured 1.8-fold to 4.4-fold more trash than the walkthrough method alone. Ground truth validation of UAV-identified trash showed that 94% of the labeled trash sites were correctly identified with regards to both location and trash classification. In addition, 98% of the visible trash mimics documented during walkthroughs were correctly avoided during UAV image analysis. We identified advantages and limitations to using UAV imaging to identify trash piles. While UAV imaging did miss trash underneath vegetation or buildings and did not show the exact composition of trash piles, this method was efficient, enabled detailed quantitative trash data, and granted access to areas that were not easily accessible by walking.

**Conclusions:**

UAVs provide a promising method of trash mapping and classification, which can improve research evaluating trash as a risk factor for infectious diseases or aiming to decrease community trash exposure.

**Graphical Abstract:**

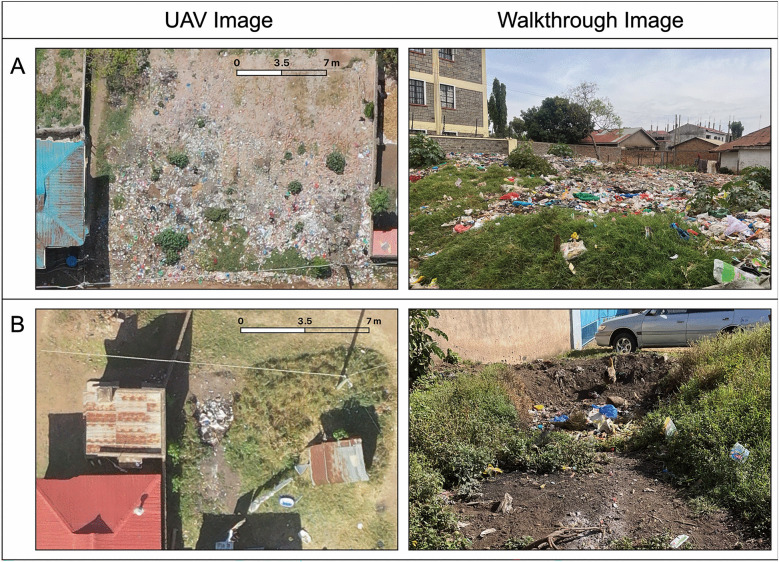

## Background

The geographic range and incidence of dengue continues to grow annually, with more than half of the global population currently at risk [1]. In 2019, 5.2 million dengue cases were reported to the World Health Organization, with total infection numbers estimated to be closer to 390 million per year [2, 3]. The *Aedes aegypti* mosquito is the primary vector for dengue, chikungunya, and Zika viruses, making it an important target for infectious disease mitigation strategies. *Ae*. *aegypti* have already been identified in 167 countries, and their endemic zone is expected to increase 10–30% by the end of the century on the basis of climate change projections [4]. They thrive in urban tropical and subtropical climates owing to climate, predator reduction, proximity to human hosts, and ideal habitat creation; in particular, water storage containers, discarded tires, and trash that fill with rain water provide ideal breeding grounds for these mosquitoes [5–13].

The rate of global plastic production and disposal grows annually, with a projected 60% of all plastics ending up in landfills or the natural environment [14]. With increased waste, and without extensive waste collection services, trash accumulates outside and is exposed to the elements. Rain and flooding fill plastic containers and abandoned tires with water that remains stagnant, a hospitable *Ae*. *aegypti* breeding ground [8, 11, 13, 15–18]. Amplified by the fact that household trash piles and community dumps are often located adjacent to populated areas for convenience, spikes in *Ae*. *aegypti* populations can lead to significant surges in arbovirus transmission [19–22]. Conversely, implementing waste collection interventions can decrease the prevalence of *Ae*. *aegypti* pupae around households [23, 24].

While the connection between trash and vector prevalence is well documented, the distribution of trash throughout a community remains difficult to quantify. In some cases, trained researchers or health workers walk through neighborhoods recording the binary presence or absence of trash [25], counting individual containers [8], or surveying participants to gather data about trash in proximity to households [26, 27]. These processes are labor and time intensive, lack detailed quantitative information, and are difficult to perform frequently or over large areas. The lack of a reliable, reproducible way to quantify the amount of trash in an area also impedes rigorous research evaluating the impact of trash interventions.

Unmanned aerial vehicles (UAVs) are increasingly being utilized to study environmental drivers of disease [28–31]. UAVs can collect much higher resolution images than satellites, and flights can be deployed at specific intervals, enabling researchers to evaluate environmental variables at finer spatial and temporal scales. In contrast to walking through a study site, UAVs can survey a significantly larger region in a fraction of the time [32]. Using UAV imaging to identify mosquito habitats has been piloted in various infectious disease applications, including mapping surface water bodies [33], high risk land cover [34–36], and household water containers [37]. Quantifying trash with UAV imaging has been piloted in the context of onshore and offshore marine waste [38–40], individual container types [41], and illegal dumping sites [42]. However, the application of UAV-derived imaging to identify and quantify trash through the lens of *Ae*. *aegypti* breeding habitat risk remains limited.

Although the development of UAV technology presents an opportunity for efficient, versatile, and detailed quantification of trash distribution, potential limitations include visual artifacts distorting regions of the image, inaccurate trash classification or reviewer bias, and visual obstacles obscuring trash from the aerial view [43]. The objective of this study is to evaluate the efficacy of using UAVs for the identification and classification of trash piles that could serve as *Ae*. *aegypti* breeding sites by comparing UAV imaging with traditional walkthroughs in two communities in Kenya where trash has been linked to arboviral exposure risk [8, 11, 12, 16, 44].

## Methods

### Study site

The UAV flights and walkthroughs took place in two sites in Kenya: Kisumu City and the town of Ukunda. The two sites exhibit distinct climate and topographical features, with Kisumu characterized by its urban inland environment and Ukunda by its semi-urban coastal setting. These variations impact trash accumulation patterns and mosquito habitat characteristics. The study sites were selected on the basis of the prevalence of trash accumulation and documented incidences of dengue and chikungunya, ensuring the relevance of the findings to high-risk areas [8, 11–13, 16, 45–62].

### Trash classification

Images obtained from UAV flights were reviewed for the presence of trash with manual visual inspection, and each trash pile was placed in a category aligned with the classification scheme. Trash was identified and categorized by a trained individual using a trash classification scheme that we previously developed for these sites [31]. This scheme accounts for the visual appearance of trash and assigns a risk score to each trash category on the basis of factors that would impact the likelihood of the trash site being a productive *Ae*. *aegypti* breeding ground [31]. Trash categories included: trash collection center, large community dump, medium community dump, small household trash pile, trash pile next to water canal, discarded car tire, mixed trash and rubble, scattered trash in the grass, and scattered trash by the road. The broader label of “higher risk trash” was assigned to trash collection centers, large community dumps, and medium community dumps because the size, density, and level of disturbance of these categories make them more likely to serve as good *Aedes aegypti* breeding sites [31].

**Fig. 1:**
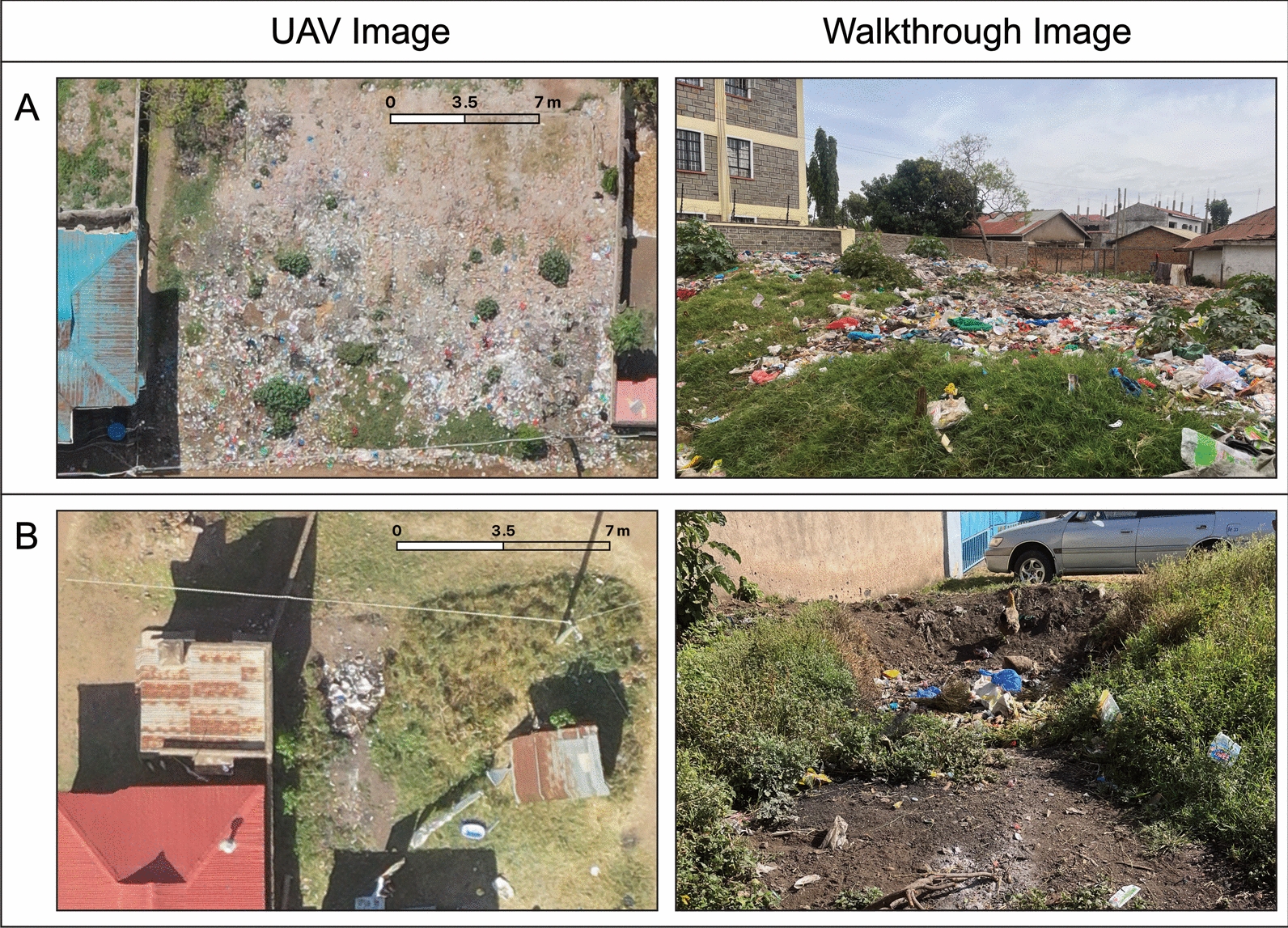
Comparison of UAV and walkthrough images. Trash piles visible on the UAV images are shown in a side-by-side comparison with photos taken during walkthroughs. These trash sites are classified as (**A**), a large community dump with “higher risk trash” and (**B**), a lower risk, household trash pile. Comparison of UAV images and walkthrough photos of the complete trash classification scheme has previously been described [31]. *UAV* unmanned aerial vehicle

Using QGIS version 3.24, a 5 × 5 m grid was applied to the UAV image and each grid square was inspected by visual review to ensure that all sections of the map were addressed. A polygon was drawn around each trash pile identified on the UAV image, and each polygon was individually classified by trash category. During community walkthroughs, trash was observed in person and categorized according to the same classification scheme. The trash location and classification were compared between the UAV images and the walkthrough data (Fig. 1).

### UAV flight planning and image acquisition

Flights were conducted using a DJI Mavic 2 UAV in collaboration with SwiftLabs (https://swiftlab.tech/) and in accordance with Kenyan Aviation Authority regulations. Flights were conducted at an altitude of approximately 100 m over 8 days in January 2023 and August 2023.

### UAV image processing

Image processing was done using AgiSoft Metashape Professional version 1.8.4 and base maps were generated in geographic coordinate system WGS 84 with a resolution of 3 cm per pixel, and exported as a geotiff.

### Four types of community walkthrough methods

**Fig. 2:**
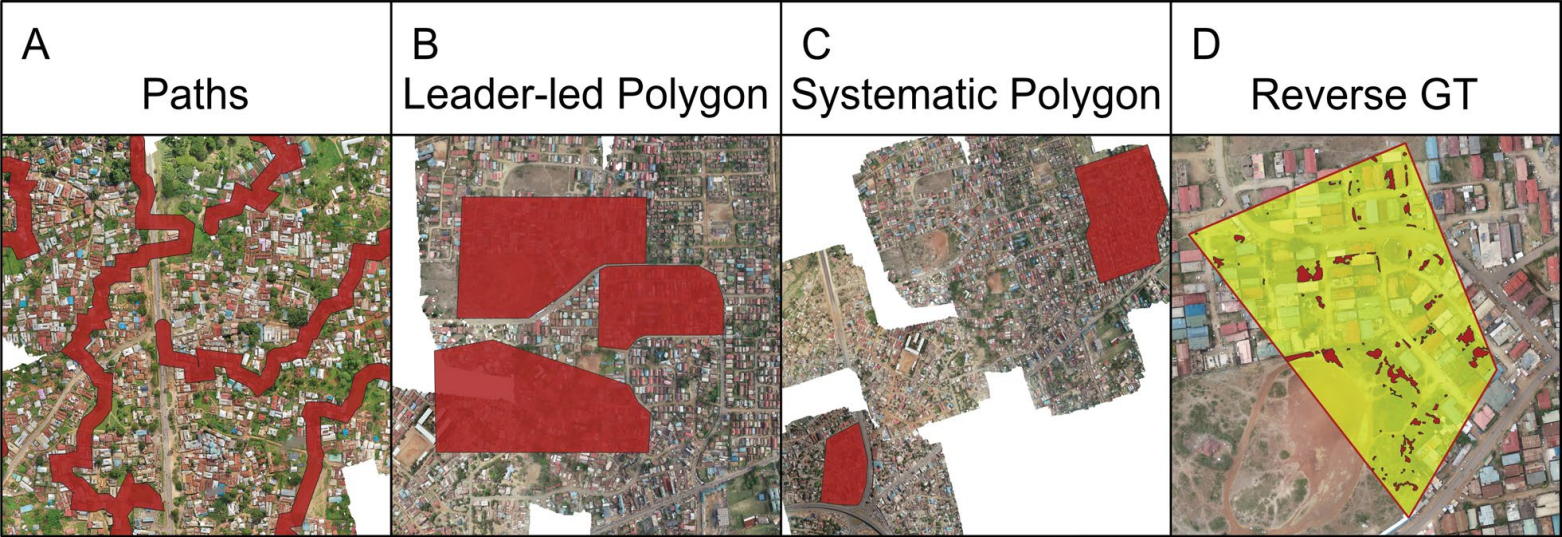
Walkthrough methods. **A**, A section of the path walkthrough route through Ukunda, with a 15-m buffer shown in red to represent land covered by visual inspection. **B**, The three red polygons outline the regions in Kisumu where leader-led walkthroughs were conducted. **C**, Two areas in Kisumu that were systematically examined for all visible trash during the walkthrough. **D**, The reverse ground truthing area in Kisumu, with trash points labeled via UAV image shown in red. *GT* ground truth

To provide ground truth data to compare with the UAV image data, we conducted walkthroughs of the communities through which trash sites were identified by visual inspection on the ground and their location and trash type were recorded. Although walkthrough-based trash counting is frequently used to observe trash distribution, this study took a unique and iterative approach to the walkthrough process; we employed four distinct approaches to improve the reliability of walkthroughs as a ground truthing method for UAV trash identification. Initially, we used exploratory walkthroughs of the environment to highlight potential obstacles to UAV imaging and to determine which common objects found in the study site region mimicked trash piles when viewed from an aerial perspective. As information was gathered, we fine-tuned the walkthrough process; ultimately, four methods of walkthroughs were developed and used across the two study sites (Fig. 2). Walkthroughs occurred on the same day as the UAV flight for a given area or, when not possible, within 2 days.

s

1. Paths: general trash site identification along major walking paths (Ukunda, January 2023)

Initial walkthroughs followed a route that covered 7 km and was distributed throughout the study site. Using an eTrek10 GPS device and a smartphone, trained study staff marked the trash coordinates, noted trash classification details, and took a photo of high and medium risk trash along the route, documenting trash site characteristics including size and category. Additionally, non-trash objects and piles that looked similar to trash were marked and labeled as a mimic. Post-walkthrough, a 15-m buffer was overlaid on the walking route with QGIS to represent the visual field from the path for analysis purposes, resulting in a total walkthrough coverage area of 0.21 square km (sqkm). The buffer distance was determined by reviewing the UAV image and accounting for the visual obstruction of houses along the path.

2 Leader-led polygon: identification of major trash sites within a community boundary (Kisumu, January 2023)

Community leaders and trained study staff walked through a 0.33 sqkm region of Kisumu with the goal of finding all large and medium trash sites well-known to the local community leaders. Smaller trash sites including small household piles, scattered trash, and tires were also documented throughout, although not with the same level of detail as was used in the systematic walkthrough described below.

3 Systematic polygon: identification of every trash site within an established boundary (Kisumu, January 2023)

Trained study staff walked systematically through a 0.23 sqkm region delineated by a predefined boundary polygon defined on QGIS and uploaded to maps. Study staff walked through the area guided by the Google Maps boundary with the intent of identifying every trash site, regardless of size, within the study area. All visible trash points were marked, along with a description of the trash classification and a photo.

4 Reverse ground truthing: verification of trash identity post-UAV image processing (Kisumu and Ukunda August 2023)

Immediately following the UAV flight and initial map processing, one trained individual reviewed the UAV images and identified trash points within an area determined to be representative of the region and containing a variety of trash types, given the broader context of previous flights—0.03 sqkm in Ukunda and 0.04 sqkm in Kisumu. The trash points and ground truthing areas were labeled on Google Maps, which was used by trained team members to complete the reverse ground truthing process, that is: 10–12 days after the UAV flight, trash points that were identified on the UAV image were visited by the team member on-site to confirm or correct the trash location and categorization.

### Trash mimics

Throughout the path and leader-led walkthroughs, the study team recorded objects and areas that could conceivably be mistaken as trash from an aerial perspective owing to similar coloring, size, and texture. These trash mimics included construction supplies, such as bricks, tarps, and rubble, as well as organic materials, such as leaves and wood, and miscellaneous objects, including livestock and fabric [31]. Mimics, unlike trash and tires, do not collect water as effectively and thus do not confer a significant risk of *Ae*. *aegypti* breeding.

### Analysis

Individual associations between the walkthrough trash points and those found with UAV imaging were determined by referencing GPS data, photos captured during the walkthroughs, and the UAV map. Image and GPS analysis was initially conducted using QGIS version 3.24 and later processed with R version 4.4.0 to aggregate and summarize the data.

### Assessment of UAV imaging advantages and disadvantages

The relative advantages and disadvantages of identifying *Ae*. *aegypti* breeding sites by UAV imaging versus community walkthroughs were then qualitatively evaluated by the study team on the basis of four criteria: visualization, spatial accuracy, temporal accuracy, and logistics.

## Results

### Trash identification by walkthroughs versus UAV method

**Fig. 3:**
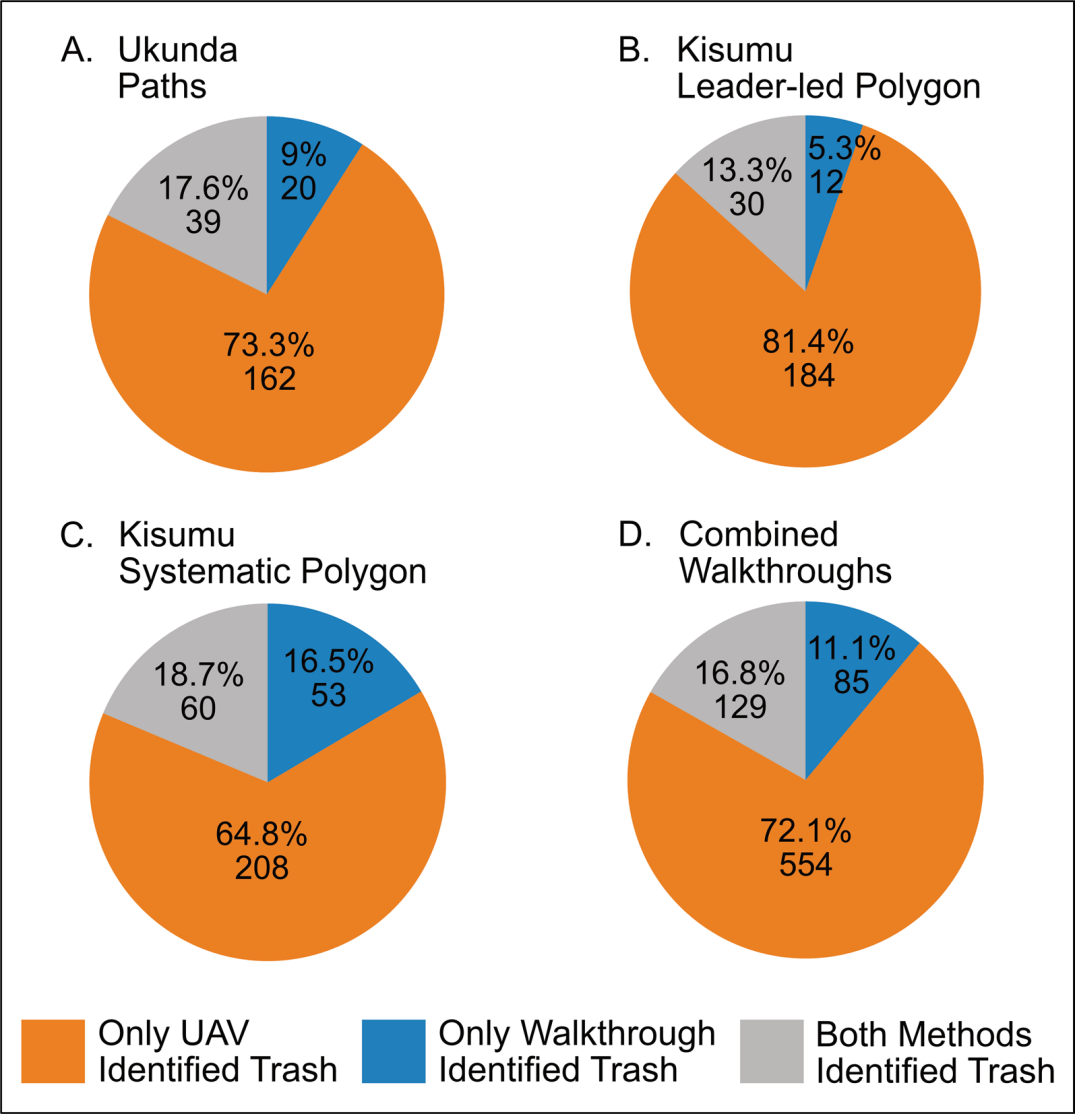
Overall trash identification by method. **A–D**, The ratio of trash identified by only the UAV, only on walkthrough, or by both methods is shown for each walkthrough method. Across all methods, UAV imaging captured more trash sites than walkthroughs and only missed 11.1% of total trash identified overall. *UAV* unmanned aerial vehicle

Together, all “forward” walkthrough methods—paths, leader-led, and systematic—identified and classified 214 trash features across all nine trash categories, 18% (39) of which fall into a “higher risk trash” category owing to their size and density: trash collection centers, large community dumps, and medium community dumps. UAV image analysis of the same regions marked 60% (129/214) of the trash points identified during the community walkthroughs. Although the UAV method did not successfully identify all of the walkthrough trash features specifically, the UAV technique identified threefold more trash than the walkthrough method within the walkthrough regions. The magnitude of this increase ranges from 1.8-fold to 4.4-fold when stratified by the walkthrough method employed in the area. However, the distributions of trash captured by each method follow a similar pattern; trash identified only with UAV imaging constitutes the largest percentage, followed by trash identified by both methods and trash found only on walkthrough (Fig. 3).

### Identification by trash categories

The most common trash category documented was small household piles, followed by scattered trash and discarded car tires. Higher risk trash categories accounted for less than one tenth of the total trash found by both UAV and walkthrough methods combined. Within this smaller subsection of trash, the UAV method identified 82% (32/39) of the higher risk piles that were found on walkthrough and contributed 27 additional higher risk piles—all of which fell under the classification of medium community dumps (Table 1). Of the seven high-risk piles missed by UAV, two were large community dumps hidden under features that obscured the aerial view: a dump under the canopy of a large fig tree and a dump within a covered building.

**Table 1:** Trash feature identification stratified by method and trash category

Trash category	Ukunda paths				Kisumu leader-led polygon				Kisumu systematic polygon				Combined all walkthroughs			
	Both	Only UAV	Only WT	Total	Both	Only UAV	Only WT	Total	Both	Only UAV	Only WT	Total	Both	Only UAV	Only WT	Total
Total (all trash categories)	39 (18%)	162 (73%)	20 (9%)	221	30 (13%)	184 (81%)	12 (5%)	226	60 (19%)	208 (65%)	53 (17%)	321	129 (17%)	554 (72%)	85 (11%)	768
Trash collection center*	3 (100%)	0 –	0 –	3	1 (100%)	0 –	0 –	1	0 –	0 –	0 –	0	4 (100%)	0 –	0 –	4
Large community dump*	5 (83%)	0 –	1 (17%)	6	2 (67%)	0 –	1 (33%)	3	0 –	0 –	0 –	0	7 (78%)	0 –	2 (22%)	9
Medium community dump*	12 (46%)	13 (50%)	1 (4%)	26	5 (31%)	11 (69%)	0 –	16	4 (36%)	3 (27%)	4 (36%)	11	21 (40%)	27 (51%)	5 (9%)	53
Small household trash pile	11 (12%)	70 (79%)	8 (9%)	89	17 (15%)	89 (79%)	7 (6%)	113	28 (20%)	86 (61%)	26 (19%)	140	55 (16%)	245 (72%)	41 (12%)	342
Trash pile next to water canal	0 –	0 –	1 (100%)	1	0 –	0 –	0 –	0	3 (14%)	15 (68%)	4 (18%)	22	3 (13%)	15 (65%)	5 (22%)	23
Discarded car tire	1 (6%)	13 (72%)	4 (22%)	18	0 –	21 (95%)	1 (5%)	22	4 (7%)	50 (82%)	7 (11%)	61	5 (5%)	84 (83%)	12 (12%)	101
Mixed trash and rubble	1 (13%)	5 (63%)	2 (25%)	8	1 (14%)	3 (43%)	3 (43%)	7	1 (33%)	0 –	2 (67%)	3	2 (12%)	8 (47%)	7 (41%)	18
Scattered trash in the grass	4 (8%)	45 (88%)	2 (4%)	51	2 (4%)	50 (96%)	0 –	52	16 (29%)	35 (64%)	4 (7%)	55	20 (13%)	131 (83%)	6 (4%)	158
Scattered trash by the road	2 (11%)	16 (84%)	1 (5%)	19	2 (17%)	10 (83%)	0 –	12	4 (14%)	19 (66%)	6 (21%)	29	8 (13%)	45 (75%)	7 (12%)	60

For each walkthrough method, the trash points are shown categorized by trash type and separated by whether both or only one of the UAV and walkthrough methods successfully identified the trash, regardless of labeled classification. Overall, review of UAV images consistently identified more trash sites than the walkthroughs across all walkthrough methods.

*Indicates a “higher risk trash” category

*UAV* unmanned aerial vehicle, *WT* walkthrough

Discarded car tires accounted for 13% of the total trash found by both methods. Although the UAV method identified less than a third (5/17) of the car tires that were documented during the walkthroughs, the UAV also found an additional 84 tires that were missed by the walkthroughs—more than a fivefold increase in the total number of tires identified.

Among all classification categories, 45% (38/85) of the trash points that were identified by walkthrough but missed with the UAV were not visible on the UAV image owing to visual obstructions. In addition to tree canopies and roofs, obstructions included awnings and vegetation.

### Reverse ground truthing (UAV identification validation)

Using the reverse ground truthing method, 161 trash points were identified on UAV images and subsequently evaluated on-site with targeted ground truthing. These points were distributed between study sites—69 piles in the Ukunda site and 92 in Kisumu. Combined analysis of both sites concluded that 94% (151/161) of the UAV-identified trash was verified as correct with regards to both location and classification. Six of the discordant trash features were objects moved before ground truthing occurred. Specifically, six adjacent tires lying horizontally in Ukunda were marked as abandoned tires on the UAV image but were not present at the time of the ground truth, 10–12 days later; we then verified that those tires had been moved in the intervening days out of the field of view. There were four classification errors in Ukunda as well: one pile of stones was incorrectly labeled as a small trash pile, and three areas that were labeled as small trash piles were, through ground truthing, determined to be completely burned ground. A fully burned area, evidence of a previous trash pile, does not have any trash present to hold water and therefore does not pose a mosquito breeding risk.

### Mimics analysis

There were 75 trash mimics, such as organic and building materials, that were documented during the walkthroughs. These included 14 mimics that were not visible on the UAV images owing to visual obstructions (Table 2). Of the mimic points that were visible, the UAV method correctly avoided 98% (60/61)—not identifying these lookalikes as trash. The one mimic mistakenly identified as trash was an additional instance of an area of burned ground where a trash pile had been previously, but which did not contain trash capable of holding water.

**Table 2:** Identification of trash mimics

	Ukunda January 2023	Kisumu January 2023	Total (both sites)
Total mimics identified via walkthrough	47	28	75
Correct (mimics not identified as trash on UAV image)	38	22	60 (80%)
Incorrect (mimics identified as trash on UAV image)	0	1	1 (1.3%)
Not visible on UAV image	9	5	14 (18.7%)

Various objects or environmental features were predicted on walkthrough to potentially resemble trash from an aerial perspective and thus were identified as potential trash mimics [31]. These mimics pose no risk of *Aedes aegypti* breeding and identification of these features as trash would be incorrect. For each mimic identified on walkthrough, the associated UAV image location was crosschecked to determine the presence or absence of a trash feature outlined on QGIS, or if the feature was obscured from the aerial view and could not be seen.

*UAV* unmanned aerial vehicle

### Qualitative comparison of UAV imaging versus walkthrough approaches

In addition to the quantitative comparison of the number of trash sites identified by UAV imaging versus walkthrough, we identified four broad categories of qualitative factors that warrant consideration in determining whether UAV imaging may be an optimal method for a particular study question or study site. These categories included: visualization, spatial accuracy, temporal accuracy, and logistics (Table 3).

**Table 3:** Advantages and limitations of mapping solid waste with UAV imaging

Qualitative comparison of UAV versus walkthrough for trash identification:		
	Advantages of UAV imaging	Limitations of UAV imaging
Visualization	Resolution down to 3 cm per pixel, depending on flight altitude Views of hard-to-reach places (e.g., far from footpaths, inside yards or half-built buildings, and rooftops)	Cannot identify composition of trash Items under trees or eaves may be obscured Potential for misclassification
Spatial accuracy	Can quantify the surface area of trash Can get a crude estimate of trash volume based on trash density Measure geospatial epicenter and extent of trash Improved measures of places without trash	Precision of area is limited because trash boundaries are usually not discrete Cannot determine precise trash volume because view is two-dimensional
Temporal accuracy	Quantification of trash enables monitoring of distribution and overall quantity over time	Cannot determine frequency of turnover of trash within a particular pile
Logistics	Efficiently cover a relatively large area Can retrospectively review other habitats or environmental factors deemed to be of interest	Requires trained, licensed pilots to fly Review of images is time intensive Flights susceptible to disruption by weather Community may have concerns about purpose of flights

There are several factors to be considered when selecting between using a UAV image versus walkthrough approach to map trash distribution across a community. Choosing one method over the other, or combining strategies, depends on the specific research needs and the local context.

*UAV* unmanned aerial vehicle

## Discussion

This study evaluated the performance of UAV imagery compared with ground truth walkthroughs in the identification and classification of trash to map and quantify the distribution of potential *Ae*. *aegypti* breeding grounds. Comparing the UAV process with the conventional walkthrough method of trash counting showed that the aerial perspective effectively provided visuals of a significant number of trash piles that were not seen during the on-site walkthrough. Notably, UAV imaging identified additional medium community dumps, which pose a high risk of infectious disease transmission owing to their size and trash density. Although the UAV method missed approximately 11% of the trash found in the walkthrough regions, there were more than three times as many trash sites identified using the UAV as compared with the walkthroughs alone. Furthermore, on reverse ground truthing and mimic analysis, we found that UAV trash identification had an extremely low rate of misidentifying something as trash that was in fact something else.

Our findings corroborate other studies that have demonstrated the potential utility of using UAV imaging to identify containers that serve as mosquito breeding grounds or to identify trash. However, our study adds a unique perspective by focusing on terrestrial trash that can serve as *Ae*. *aegypti* breeding sites. The successful use of UAVs to monitor trash in and around oceans and rivers has been well documented [38, 39, 63–66]. However, while water and sand provide relatively neutral and consistent backdrops for visual trash identification, diverse land cover can make accurate trash quantification more difficult [41, 43]. Despite this challenge, manual review of the UAV images in this study proved highly accurate in discriminating between trash and objects that mimic trash. In a trash visualization study in South Africa, Swanepoel et al. addressed a similar research gap related to the quantification of trash sites over a large land area using UAV imagery. They found that UAVs were a successful method, although their study did not classify trash through an infectious disease lens, nor did it implement a ground truthing component [43]. In terms of container identification for mosquito habitat mapping, a study using UAV imagery to measure *Aedes albopictus* breeding risk determined that the UAV was unable to identify all of the target water containers. However, the increased efficiency of UAV technology as compared with walkthroughs was hugely beneficial—the UAV was able to assess five times the number of houses in around one tenth of the time [32]. A second study, conducted in China, found the UAV method to be almost perfectly successful at *Ae*. *albopictus* container identification, with 97% accuracy [67]. This work has built a foundation for the use of UAVs as a tool for both trash and infectious disease mapping applications. Our study adds to this growing field by demonstrating the feasibility and potential advantages of using UAV imaging to map trash types that serve as potential *Ae*. *aegypti* breeding sites.

Studies piloting the use of UAVs for mosquito breeding ground mapping generally agree that a combination of survey approaches, including UAV and manual walkthroughs, would provide the most accurate picture of risk distribution [32, 37, 68]. While other studies focus on the risk of standalone containers, such as water jugs and collection bowls, our study similarly found that the combination of UAV imaging and walkthroughs would identify the largest number of potential trash breeding grounds. Previous studies using UAVs to identify individual containers have had variable success, concluding that walkthroughs are typically more comprehensive and reliable for trash quantification [37, 41, 69]. By contrast, in this study, UAV image analysis found a much greater quantity of trash than manual walkthroughs alone, suggesting the increased value of the aerial perspective when the target objects are trash piles and tires rather than smaller scale objects. Building on a wealth of similar studies adopting UAV imaging to assist with pollution, infectious disease, and environmental management projects, this study addresses a novel application of UAV technology in its scale, vector focus, and region. Uniquely, this study uses UAVs to locate and categorize trash through the lens of *Ae*. *aegypti* breeding risk, rather than identifying environmental hotspots or individual containers.

A key limitation of UAV imaging is the lack of visibility when trash sites are located under tree canopies or within covered structures, a finding consistent with studies analyzing UAV applications [32, 41, 68]. Features completely hidden from the aerial perspective accounted for 38/85 (44.7%) of the trash that was identified by walkthrough but missed on the UAV image. Although this subset of hidden trash represents a meaningful portion of the trash sites, one way to mitigate this limitation is to use subcategories that indicate when a pile appears to be partially hidden from view. In our previous study, we proposed subcategories such as “under a tree” and “mixed with vegetation,” as these are common features that obstruct the full visualization of a trash pile and suggest that the pile is likely larger in size and therefore higher risk than the measured size would suggest [31]. This approach does not completely negate the possibility of missing piles entirely obstructed from view, but can help mitigate this problem. Moreover, trash and tires located under roofs and building cover are often protected to some degree from rainwater and may serve as a less productive breeding site owing to the reduced quantity of collected water. Future studies could explore the integration of LiDAR technology with UAV imaging to overcome limitations posed by visual obstructions, such as tree canopies or building roofs.

Classification subcategories can further be used, as proposed by Rosser et al., to address ambiguity in trash sites that have been burned and may or may not still serve as productive breeding ground, which can be difficult to discern with UAV imaging. Of the 11 features that were found to be incorrectly classified across the reverse ground truthing and mimics analysis, four mistakes were based on the differentiation between a partially and fully burned area. The subcategory “burn” is useful to understand if there is a high disturbance rate of the trash—which corresponds to a lower risk of mosquito breeding—as areas are often used repeatedly to burn household trash. Therefore, the distinction between the extent to which the trash has been burned may not be critical, as all trash with evidence of burning is lower risk. The remaining reverse ground truthing misses were a result of tires that were moved between UAV flight and ground truthing; these features reflect more the challenge of predicting trash movement patterns than the efficacy of UAVs for trash identification itself.

Our reverse ground truth accuracy was limited by delays between drone flights, map processing, image analysis, and ground truth verification in the field. In that 10–12 day interval, we did find that several of the tires had been moved. If it had been possible to conduct the drone flights and ground truth walkthroughs on the same day, it is likely that our reverse ground truth accuracy would improve. This observation also highlights the variability of trash movement by trash type; tires and containers are more mobile than other classifications, such as large, stationary dump sites. However, the challenge of mapping dynamic breeding sites is consistent across both UAV and walkthrough methods.

In some areas, reviewers faced challenges distinguishing trash from the background image, which led to the under-identification of trash using the UAV method. Vegetation providing partial cover, low contrast with the background colors, resemblance to rocks or plant matter, or overexposure and shadowing artifacts from the UAV camera made certain trash areas difficult to identify, as has also been seen in other studies [38, 43, 64, 69]. This challenge impacted the identification of smaller trash and large dumps to a similar degree, suggesting that a finer pixel resolution of the UAV image would not necessarily improve the percentage of trash capture.

Despite these limitations, the aerial perspective and ability to visualize areas far from walking paths allowed the UAV images to reveal dumps in overgrown areas and trash piles behind walled courtyards, among other areas generally inaccessible by foot. Consistent across all walkthrough methods, the significant increase in tire identification using the UAV method indicates that tires are uniquely suited for UAV imaging. Their consistent shape and high contrast with the ground enable easy identification, and because of the frequency of tire storage on roofs and in areas not visible from the ground, the aerial perspective is especially useful. UAV imaging can not only facilitate higher detection rates but also significantly reduce the time and resources required for large-scale trash mapping compared with traditional walkthroughs. The timeline of UAV image analysis also allows the reviewer to take breaks and return to the trash identification task as needed after the initial UAV images are taken. In contrast, the walkthrough method is more labor-intensive, and the identification process cannot be segmented, replicated, or reviewed later. Furthermore, the UAV images can be used to develop machine learning algorithms for automatic trash recognition, a step that will streamline the identification process, improving consistency and efficiency, and allow for serial assessments and the evaluation of larger areas. In addition to the increased number of trash sites identified, UAV technology generates detailed quantitative data about trash surface area. Surface area data is difficult to accurately measure with a walkthrough, but it is valuable information because of the range of pile sizes within one trash classification category.

One limitation of this study was the comparison of the UAV method with ground truthing walkthroughs. The walkthrough method does not capture the entirety of trash in the region and is therefore a poor gold standard. To account for this fact, we used multiple walkthrough approaches—three methods to evaluate the ability of the UAV to capture all trash in a given area, and two approaches to evaluate the accuracy of the UAV method in identifying trash and not mislabeling mimics as trash. We found similar results across these different methods, suggesting that in future studies, selecting a single forward ground truth approach and a single reserve ground truth approach may be sufficient when evaluating a new site. However, the approaches were optimized for assessment of different types of trash, and thus, the highest priority trash targets should be considered when selecting a ground truth approach. Another limitation of this study was the use of only two study sites, both in Kenya with relatively similar climates and housing construction. Despite these similarities, our sites did have some notable differences: one was an urban, inland site and the other was a semi-urban, coastal site. Moreover, while brick piles were found to be common mimics in both sites, the differences in the type of earth in the two regions altered the color of these bricks and thus their appearance on the UAV images. The fact that we found similar results and good overall UAV performance across both sites suggests that our findings could be applicable to many settings. Climate change will increase the abundance and geographic spread of *Ae*. *aegypti* mosquitos, thus requiring more effective strategies for vector habitat mitigation [70]. As UAV imaging as a method of mapping vector breeding habitats expands, it is critical to evaluate the accuracy and potential pitfalls of adapting this methodology to new locations with different soil, climate, and construction features.

Efficiency and access are major advantages of UAV imaging as a tool for vector habitat mapping and risk assessment. While the UAV method showed improved trash identification in the two Kenyan field sites explored in this study, applications of this technology to more difficult-to-reach locations may be even more impactful. UAVs facilitate the quantification of *Ae*. *aegypti* habitat in areas where walkthroughs are not possible, enabling the implementation of vector abatement strategies and study of the dynamics between infectious disease and environmental factors. This technology has the potential to increase the reach and efficacy of existing public health resources, as well as provide improved data collection as a foundation for innovation.

## Conclusions

Comparable or superior trash identification, accurate classification, and more detailed quantification, relative to walkthroughs, suggest that UAV technology is a promising method of trash mapping for infectious disease applications. A combination of UAV imaging and community walkthroughs may provide the most complete catalog of the trash in an area, especially when working with community leaders who can ensure that known dumps and high-risk sites are not overlooked. This study supports the application of UAV imaging to map and quantify trash that serves as high-risk *Ae*. *aegypti* habitat. This tool can be applied for a variety of uses, such as quantifying changes in trash over time, across different seasons, or before and after an intervention.

## Data Availability

Aerial images will be made available to researchers who provide a methodologically sound proposal. Proposals should be directed to jrosser@stanford.edu; to gain access, data requestors will need to sign a data access agreement.

## References

[1] Messina JP, Brady OJ, Golding N, Kraemer MUG, Wint GRW, Ray SE, et al. The current and future global distribution and population at risk of dengue. Nat Microbiol. 2019;4:1508–15. 31182801 10.1038/s41564-019-0476-8 PMC6784886

[2] Bhatt S, Gething PW, Brady OJ, Messina JP, Farlow AW, Moyes CL, et al. The global distribution and burden of dengue. Nature. 2013;496:504–7. 23563266 10.1038/nature12060 PMC3651993

[3] World Health Organization. Dengue and severe dengue [Internet]. World Health Organ. 2023 [cited 2024 Apr 18]. Available from: https://www.who.int/news-room/fact-sheets/detail/dengue-and-severe-dengue.

[4] Laporta GZ, Potter AM, Oliveira JFA, Bourke BP, Pecor DB, Linton Y-M. Global distribution of *Aedes aegypti* and *Aedes albopictus* in a climate change scenario of regional rivalry. Insects. 2023;14:49. 36661976 10.3390/insects14010049 PMC9860750

[5] Arunachalam N, Tana S, Espino F, Kittayapong P, Abeyewickreme W, Wai KT, et al. Eco-bio-social determinants of dengue vector breeding: a multicountry study in urban and periurban Asia. Bull World Health Organ. 2010;88:173–84. 20428384 10.2471/BLT.09.067892 PMC2828788

[6] Banerjee S, Aditya G, Saha GK. Household disposables as breeding habitats of dengue vectors: linking wastes and public health. Waste Manag. 2013;33:233–9. 23107350 10.1016/j.wasman.2012.09.013

[7] Banerjee S, Aditya G, Saha GK. Household wastes as larval habitats of dengue vectors: comparison between urban and rural areas of Kolkata, India. PLoS ONE. 2015;10:e0138082. 26447690 10.1371/journal.pone.0138082 PMC4598039

[8] Ngugi HN, Mutuku FM, Ndenga BA, Musunzaji PS, Mbakaya JO, Aswani P, et al. Characterization and productivity profiles of *Aedes aegypti* (L.) breeding habitats across rural and urban landscapes in western and coastal Kenya. Parasit Vectors. 2017;10:331. 28701194 10.1186/s13071-017-2271-9 PMC5508769

[9] Wilke ABB, Chase C, Vasquez C, Carvajal A, Medina J, Petrie WD, et al. Urbanization creates diverse aquatic habitats for immature mosquitoes in urban areas. Sci Rep. 2019;9:15335. 31653914 10.1038/s41598-019-51787-5 PMC6814835

[10] Kampango A, Furu P, Sarath DL, Haji KA, Konradsen F, Schiøler KL, et al. Risk factors for occurrence and abundance of *Aedes aegypti* and *Aedes bromeliae* at hotel compounds in Zanzibar. Parasit Vectors. 2021;14:544. 34686195 10.1186/s13071-021-05005-9 PMC8539800

[11] Mwakutwaa AS, Ngugi HN, Ndenga BA, Krystosik A, Ngari M, Abubakar LU, et al. Pupal productivity of larval habitats of *Aedes aegypti* in Msambweni, Kwale County, Kenya. Parasitol Res. 2023;122:801–14. 36683088 10.1007/s00436-022-07777-0 PMC9988718

[12] Khan A, Bisanzio D, Mutuku F, Ndenga B, Grossi-Soyster EN, Jembe Z, et al. Spatiotemporal overlapping of dengue, chikungunya, and malaria infections in children in Kenya. BMC Infect Dis. 2023;23:183. 36991340 10.1186/s12879-023-08157-4 PMC10053720

[13] Peña-García VH, Mutuku FM, Ndenga BA, Mbakaya JO, Ndire SO, Agola GA, et al. The importance of including non-household environments in dengue vector control activities. Viruses. 2023;15:1550. 37515236 10.3390/v15071550 PMC10384488

[14] Geyer R, Jambeck JR, Law KL. Production, use, and fate of all plastics ever made. Sci Adv. 2017;3:e1700782. 28776036 10.1126/sciadv.1700782 PMC5517107

[15] Getachew D, Tekie H, Gebre-Michael T, Balkew M, Mesfin A. Breeding sites of *Aedes aegypti* : potential dengue vectors in Dire Dawa, East Ethiopia. Interdiscip Perspect Infect Dis. 2015;2015:1–8. 10.1155/2015/706276 PMC4576013 26435712

[16] Ngugi HN, Nyathi S, Krystosik A, Ndenga B, Mbakaya JO, Aswani P, et al. Risk factors for *Aedes aegypti* household pupal persistence in longitudinal entomological household surveys in urban and rural Kenya. Parasit Vectors. 2020;13:499. 33004074 10.1186/s13071-020-04378-7 PMC7528257

[17] Mukhtar MU, Han Q, Liao C, Haq F, Arslan A, Bhatti A. Seasonal distribution and container preference ratio of the dengue fever vector (Aedes aegypti, Diptera: Culicidae) in Rawalpindi, Pakistan. J Med Entomol. 2018;55:1011–5. 29462424 10.1093/jme/tjy010

[18] Fansiri T, Buddhari D, Pathawong N, Pongsiri A, Klungthong C, Iamsirithaworn S, et al. Entomological risk assessment for dengue virus transmission during 2016–2020 in Kamphaeng Phet, Thailand. Pathogens. 2021;10:1234. 34684183 10.3390/pathogens10101234 PMC8538081

[19] Hayes JM, García-Rivera E, Flores-Reyna R, Suárez-Rangel G, Biggerstaff BJ, Rodríguez-Mata T, et al. Risk factors for infection during a severe dengue outbreak in El Salvador in 2000. Am J Trop Med Hyg. 2003;69:629–33. 14740880

[20] Brunkard JM, López JLR, Ramirez J, Cifuentes E, Rothenberg SJ, Hunsperger EA, et al. Dengue fever seroprevalence and risk factors, Texas –Mexico Border, 2004. Emerg Infect Dis. 2007;13:1477–83. 18257990 10.3201/eid1310.061586 PMC2851499

[21] Bostan N, Javed S, Nabgha-e-Amen, Eqani SAMAS, Tahir F, Bokhari H. Dengue fever virus in Pakistan: effects of seasonal pattern and temperature change on distribution of vector and virus: increasing prevalence of dengue in Pakistan. Rev Med Virol. 2017;27:e1899. 10.1002/rmv.1899 27597296

[22] Krystosik A, Njoroge G, Odhiambo L, Forsyth JE, Mutuku F, LaBeaud AD. Solid wastes provide breeding sites, burrows, and food for biological disease vectors, and urban zoonotic reservoirs: a call to action for solutions-based research. Front Public Health. 2020;7:405. 32010659 10.3389/fpubh.2019.00405 PMC6979070

[23] Vanlerberghe V, Toledo ME, Rodríguez M, Gomez D, Baly A, Benitez JR, et al. Community involvement in dengue vector control: cluster randomised trial. BMJ. 2009;338:b1959. 19509031 10.1136/bmj.b1959 PMC2694260

[24] Abeyewickreme W, Wickremasinghe AR, Karunatilake K, Sommerfeld J, Kroeger A. Community mobilization and household level waste management for dengue vector control in Gampaha district of Sri Lanka; an intervention study. Pathog Glob Health. 2012;106:479–87. 23318240 10.1179/2047773212Y.0000000060 PMC3541909

[25] Heukelbach J, De Oliveira FAS, Kerr-Pontes LRS, Feldmeier H. Risk factors associated with an outbreak of dengue fever in a favela in Fortaleza, north-east Brazil. Trop Med Int Health. 2001;6:635–42. 11555429 10.1046/j.1365-3156.2001.00762.x

[26] Suwanbamrung C, Thoutong C, Eksirinimit T, Tongjan S, Thongkew K. The use of the “Lansaka Model” as the larval indices surveillance system for a sustainable solution to the dengue problem in southern Thailand. PLoS ONE. 2018;13:e0201107. 30067819 10.1371/journal.pone.0201107 PMC6070242

[27] Zolnikov TR, Clark T, Furio F, Yasobant S, Martins ACS, Cruvinel VRN, et al. “Look, it’s a dengue mosquito”: a qualitative study on living near open-air dumpsites and vector-borne diseases. Adv Environ Eng Res. 2023;04:1–25.

[28] Wood CL, Sokolow SH, Jones IJ, Chamberlin AJ, Lafferty KD, Kuris AM, et al. Precision mapping of snail habitat provides a powerful indicator of human schistosomiasis transmission. Proc Natl Acad Sci USA. 2019;116:23182–91. 31659025 10.1073/pnas.1903698116 PMC6859407

[29] Chamberlin AJ, Jones IJ, Lund AJ, Jouanard N, Riveau G, Ndione R, et al. Visualization of schistosomiasis snail habitats using light unmanned aerial vehicles. Geospatial Health. 2021;15. Available from: https://geospatialhealth.net/index.php/gh/article/view/818. 10.4081/gh.2020.818 33461284

[30] Jones IJ, Sokolow SH, Chamberlin AJ, Lund AJ, Jouanard N, Bandagny L, et al. Schistosome infection in Senegal is associated with different spatial extents of risk and ecological drivers for *Schistosoma haematobium* and *S.**mansoni*. PLoS Negl Trop Dis. 2021;15:e0009712. 34570777 10.1371/journal.pntd.0009712 PMC8476036

[31] Rosser JI, Tarpenning MS, Bramante JT, Tamhane A, Chamberlin AJ, Mutuku PS, et al. Development of a trash classification system to map potential *Aedes aegypti* breeding grounds using unmanned aerial vehicle imaging. Environ Sci Pollut Res. 2024;31:41107–17. 10.1007/s11356-024-33801-0 PMC11189966 38842780

[32] Case E, Shragai T, Harrington L, Ren Y, Morreale S, Erickson D. Evaluation of unmanned aerial vehicles and neural networks for integrated mosquito management of *Aedes albopictus* (Diptera: Culicidae). J Med Entomol. 2020;57:1588–95. 32474595 10.1093/jme/tjaa078

[33] Hardy A, Makame M, Cross D, Majambere S, Msellem M. Using low-cost drones to map malaria vector habitats. Parasit Vectors. 2017;10:29. 28088225 10.1186/s13071-017-1973-3 PMC5237572

[34] Landau KI, Van Leeuwen WJD. Fine scale spatial urban land cover factors associated with adult mosquito abundance and risk in Tucson. Arizona J Vector Ecol. 2012;37:407–18. 23181866 10.1111/j.1948-7134.2012.00245.x

[35] Carrasco-Escobar G, Manrique E, Ruiz-Cabrejos J, Saavedra M, Alava F, Bickersmith S, et al. High-accuracy detection of malaria vector larval habitats using drone-based multispectral imagery. PLoS Negl Trop Dis. 2019;13:e0007105. 30653491 10.1371/journal.pntd.0007105 PMC6353212

[36] Sarira TV, Clarke K, Weinstein P, Koh LP, Lewis M. Rapid identification of shallow inundation for mosquito disease mitigation using drone-derived multispectral imagery. Geospatial Health. 2020;15. Available from: https://geospatialhealth.net/index.php/gh/article/view/851. 10.4081/gh.2020.851 32575964

[37] Valdez-Delgado KM, Moo-Llanes DA, Danis-Lozano R, Cisneros-Vázquez LA, Flores-Suarez AE, Ponce-García G, et al. Field effectiveness of drones to identify potential *Aedes aegypti* breeding sites in household environments from Tapachula, a dengue-endemic city in Southern Mexico. Insects. 2021;12:663. 34442229 10.3390/insects12080663 PMC8396529

[38] Andriolo U, Gonçalves G, Rangel-Buitrago N, Paterni M, Bessa F, Gonçalves LMS, et al. Drones for litter mapping: an inter-operator concordance test in marking beached items on aerial images. Mar Pollut Bull. 2021;169:112542. 34052588 10.1016/j.marpolbul.2021.112542

[39] Andriolo U, Garcia-Garin O, Vighi M, Borrell A, Gonçalves G. Beached and floating litter surveys by unmanned aerial vehicles: operational analogies and differences. Remote Sens. 2022;14:1336.

[40] Liao Y-H, Juang J-G. Real-time UAV trash monitoring system. Appl Sci. 2022;12:1838.

[41] Schenkel J, Taele P, Goldberg D, Horney J, Hammond T. Identifying potential mosquito breeding grounds: assessing the efficiency of UAV technology in public health. Robotics. 2020;9:91.

[42] Glanville K, Chang H-C. Remote sensing analysis techniques and sensor requirements to support the mapping of illegal domestic waste disposal sites in Queensland, Australia. Remote Sens. 2015;7:13053–69.

[43] Swanepoel S, Scheckle TJ, Marlin D. Implementing land-based litter surveys through visual inspection of imagery using unmanned aerial vehicles. Environ Chall. 2023;13:100753.

[44] Forsyth JE, Mutuku FM, Kibe L, Mwashee L, Bongo J, Egemba C, et al. Source reduction with a purpose: mosquito ecology and community perspectives offer insights for improving household mosquito management in coastal Kenya. PLoS Negl Trop Dis. 2020;14:e0008239. 32392226 10.1371/journal.pntd.0008239 PMC7241847

[45] Vu DM, Banda T, Teng CY, Heimbaugh C, Muchiri EM, Mungai PL, et al. Dengue and West Nile virus transmission in children and adults in coastal Kenya. Am J Trop Med Hyg. 2017;96:141–3. 27821697 10.4269/ajtmh.16-0562 PMC5239681

[46] Waggoner J, Heath CJ, Ndenga B, Mutuku F, Sahoo MK, Mohamed-Hadley A, et al. Development of a real-time reverse transcription polymerase chain reaction for O’nyong-nyong Virus and evaluation with clinical and mosquito specimens from Kenya. Am J Trop Med Hyg. 2017;97:121–4. 28719301 10.4269/ajtmh.17-0027 PMC5508918

[47] Waggoner J, Brichard J, Mutuku F, Ndenga B, Heath CJ, Mohamed-Hadley A, et al. Malaria and Chikungunya detected using molecular diagnostics among febrile Kenyan children. Open Forum Infect Dis. 2017;4:oFX110. 28702473 10.1093/ofid/ofx110 PMC5505337

[48] Vu DM, Mutai N, Heath CJ, Vulule JM, Mutuku FM, Ndenga BA, et al. Unrecognized dengue virus infections in children, Western Kenya, 2014–2015. Emerg Infect Dis. 2017;23:1915–7. 29048283 10.3201/eid2311.170807 PMC5652413

[49] Ndenga BA, Mutuku FM, Ngugi HN, Mbakaya JO, Aswani P, Musunzaji PS, et al. Characteristics of *Aedes aegypti* adult mosquitoes in rural and urban areas of western and coastal. PLoS ONE. 2017;12:e0189971. 29261766 10.1371/journal.pone.0189971 PMC5736227

[50] Grossi-Soyster EN, Cook EAJ, de Glanville WA, Thomas LF, Krystosik AR, Lee J, et al. Serological and spatial analysis of alphavirus and flavivirus prevalence and risk factors in a rural community in Western Kenya. PLoS Negl Trop Dis. 2017;11:e0005998. 29040262 10.1371/journal.pntd.0005998 PMC5659799

[51] Gudo ES, Ali S, António VS, Chelene IR, Chongo I, Demanou M, et al. Seroepidemiological studies of arboviruses in Africa. Adv Exp Med Biol. 2018;1062:361–71. 29845545 10.1007/978-981-10-8727-1_25

[52] Hortion J, Mutuku FM, Eyherabide AL, Vu DM, Boothroyd DB, Grossi-Soyster EN, et al. Acute flavivirus and alphavirus infections among children in two different areas of Kenya, 2015. Am J Trop Med Hyg. 2019;100:170–3. 30457092 10.4269/ajtmh.18-0297 PMC6335892

[53] Heath CJ, Grossi-Soyster EN, Ndenga BA, Mutuku FM, Sahoo MK, Ngugi HN, et al. Evidence of transovarial transmission of chikungunya and dengue viruses in field-caught mosquitoes in Kenya. PLoS Negl Trop Dis. 2020;14:e0008362. 32559197 10.1371/journal.pntd.0008362 PMC7329127

[54] Shah MM, Ndenga BA, Mutuku FM, Vu DM, Grossi-Soyster EN, Okuta V, et al. High dengue burden and circulation of 4 virus serotypes among children with undifferentiated fever, Kenya, 2014–2017. Emerg Infect Dis. 2020;26:2638–50. 33079035 10.3201/eid2611.200960 PMC7588514

[55] Nosrat C, Altamirano J, Anyamba A, Caldwell JM, Damoah R, Mutuku F, et al. Impact of recent climate extremes on mosquito-borne disease transmission in Kenya. PLoS Negl Trop Dis. 2021;15:e0009182. 33735293 10.1371/journal.pntd.0009182 PMC7971569

[56] Ndenga BA, Mutuku FM, Ngugi HN, Mbakaya JO, Mukoko D, Kitron U, et al. Night time extension of *Aedes aegypti* human blood seeking activity. Am J Trop Med Hyg. 2022;107:208–10. 35640647 10.4269/ajtmh.21-0309 PMC9294705

[57] Khan A, Ndenga B, Mutuku F, Bosire CM, Okuta V, Ronga CO, et al. Majority of pediatric dengue virus infections in Kenya do not meet 2009 WHO criteria for dengue diagnosis. PLOS Glob Public Health. 2022;2:e0000175. 36962138 10.1371/journal.pgph.0000175 PMC10021889

[58] Musunzaji PS, Ndenga BA, Mzee S, Abubakar LU, Kitron UD, Labeaud AD, et al. Oviposition preferences of *Aedes aegypti* in Msambweni, Kwale County. Kenya J Am Mosq Control Assoc. 2023;39:85–95. 37270926 10.2987/22-7103 PMC10885850

[59] Vu DM, Krystosik AR, Ndenga BA, Mutuku FM, Ripp K, Liu E, et al. Detection of acute dengue virus infection, with and without concurrent malaria infection, in a cohort of febrile children in Kenya, 2014–2019, by clinicians or machine learning algorithms. PLOS Glob Public Health. 2023;3:e0001950. 37494331 10.1371/journal.pgph.0001950 PMC10370704

[60] Kiener M, Shayegh N, Nyathi SV, Ndenga BA, Mutuku FM, LaBeaud AD. Low rate of asymptomatic dengue infection detected in coastal Kenya using pooled polymerase chain reaction testing. Am J Trop Med Hyg. 2024;110:738–40. 38471167 10.4269/ajtmh.23-0650 PMC10993852

[61] Nyathi S, Rezende IM, Walter KS, Thongsripong P, Mutuku F, Ndenga B, et al. Molecular epidemiology and evolutionary characteristics of dengue virus 2 in East Africa. Nat Commun. 2024;15:7832. 39244569 10.1038/s41467-024-51018-0 PMC11380673

[62] Caldwell JM, LaBeaud AD, Lambin EF, Stewart-Ibarra AM, Ndenga BA, Mutuku FM, et al. Climate predicts geographic and temporal variation in mosquito-borne disease dynamics on two continents. Nat Commun. 2021;12:1233. 33623008 10.1038/s41467-021-21496-7 PMC7902664

[63] Geraeds M, Van Emmerik T, De Vries R, Bin Ab Razak MS. Riverine plastic litter monitoring using unmanned aerial vehicles (UAVs). Remote Sens. 2019;11:2045.

[64] Lo H-S, Wong L-C, Kwok S-H, Lee Y-K, Po BH-K, Wong C-Y, et al. Field test of beach litter assessment by commercial aerial drone. Mar Pollut Bull. 2020;151:110823. 32056615 10.1016/j.marpolbul.2019.110823

[65] Gonçalves G, Andriolo U, Gonçalves L, Sobral P, Bessa F. Quantifying marine macro litter abundance on a sandy beach using unmanned aerial systems and object-oriented machine learning methods. Remote Sens. 2020;12:2599.

[66] Taddia Y, Corbau C, Buoninsegni J, Simeoni U, Pellegrinelli A. UAV approach for detecting plastic marine debris on the beach: a case study in the Po River Delta (Italy). Drones. 2021;5:140.

[67] Yu K, Wu J, Wang M, Cai Y, Zhu M, Yao S, et al. Using UAV images and deep learning in investigating potential breeding sites of Aedes albopictus. Acta Trop. 2024;255:107234. 38688444 10.1016/j.actatropica.2024.107234

[68] Lee GO, Vasco L, Márquez S, Zuniga-Moya JC, Van Engen A, Uruchima J, et al. A dengue outbreak in a rural community in Northern Coastal Ecuador: an analysis using unmanned aerial vehicle mapping. PLoS Negl Trop Dis. 2021;15:e0009679. 34570788 10.1371/journal.pntd.0009679 PMC8475985

[69] Escobar-Sánchez G, Haseler M, Oppelt N, Schernewski G. Efficiency of aerial drones for macrolitter monitoring on Baltic sea beaches. Front Environ Sci. 2021;8:560237.

[70] Mordecai EA, Ryan SJ, Caldwell JM, Shah MM, LaBeaud AD. Climate change could shift disease burden from malaria to arboviruses in Africa. Lancet Planet Health. 2020;4:e416–23. 32918887 10.1016/S2542-5196(20)30178-9 PMC7490804

